# The Effects of Mindfulness on Glycemic Control in People with Diabetes: An Overview of Systematic Reviews and Meta-Analyses

**DOI:** 10.3390/medicines10090053

**Published:** 2023-09-07

**Authors:** Hidetaka Hamasaki

**Affiliations:** Hamasaki Clinic, 2-21-4 Nishida, Kagoshima 890-0046, Japan; h-hamasaki@umin.ac.jp; Tel.: +81-099-2503535; Fax: +81-099-250-1470

**Keywords:** mindfulness, meditation, diabetes, systematic review, randomized controlled trial, glycemic control

## Abstract

**Background:** Previous research has demonstrated the effectiveness of mindfulness interventions in improving glycemic control. By enhancing attention control, emotion regulation, and self-awareness, mindfulness shows promise in managing the lifestyle factors associated with cardiovascular disease risk. However, the impact of mindfulness on glycemic control in people with diabetes remains unclear. This overview aims to summarize the current evidence of the impact of mindfulness interventions on glycemic control in people with diabetes and propose suggestions for future research. **Methods:** The author searched electronic databases (PubMed/MEDLINE, Embase, and Cochrane Library) to identify relevant systematic reviews and meta-analyses. The current evidence regarding the effects of mindfulness on glycemic control in people with diabetes was summarized. **Results:** This review evaluated a total of five systematic reviews and meta-analyses of randomized controlled trials (RCTs). Mindfulness interventions show potential for improving glycemic control as measured by hemoglobin A1c (HbA1c) levels, as well as reducing stress, depression, and anxiety in people with diabetes. Four out of five systematic reviews and meta-analyses reported a significant reduction in HbA1c levels by approximately 0.3%. However, the available studies lacked adequate description of key characteristics of study subjects, such as body mass index, medication, and disease conditions, which are essential for assessing the impact of mindfulness on glycemic control. Moreover, there was significant heterogeneity in the intervention methods employed across the included RCTs. **Conclusions:** Mindfulness interventions are effective in improving glycemic control in people with type 2 diabetes. However, the overall quality of the reviewed studies raises uncertainty regarding the effectiveness of mindfulness as a treatment for people with diabetes. Further research is necessary to elucidate the biological effects of mindfulness on physiological, neurological, and endocrinological functions in humans.

## 1. Introduction

The prevalence of diabetes is increasing worldwide, leading to elevated risks of cardiovascular diseases (CVD) and mortality, which pose significant health challenges. The global population living with diabetes was estimated to be 529 million individuals in 2021, and the global prevalence of diabetes adjusted for age was approximately 6% [1]. It is essential for people with diabetes to engage in lifestyle modifications, including a healthy diet and regular exercise, to improve glycemic control and reduce the risks of CVD and mortality. However, maintaining a healthy lifestyle is not easy for people with diabetes due to the impact of psychological and environmental factors on their motivation for managing the condition [2]. People with diabetes need to focus on their treatment and increase their awareness of health.

Mindfulness is a state of intentional and non-judgmental attention to one’s present moment experience. It involves bringing one’s awareness to the present moment, including thoughts, feelings, bodily sensations, and the surrounding environment, with an attitude of curiosity, openness, and acceptance [3]. Mindfulness is often cultivated through various practices such as meditation, where individuals focus on the breath, bodily sensations, or a particular thought or phrase. Mindfulness is often researched for its potential benefits on mental health, well-being, stress reduction, and cognitive function [4]. Therapeutic approaches such as mindfulness-based stress reduction (MBSR) or mindfulness-based cognitive therapy (MBCT) often incorporate mindfulness practices. These interventions aim to help individuals cultivate abilities to effectively navigate their thoughts, emotions, and overall psychological well-being [5]. Furthermore, previous studies have shown that mindfulness interventions are effective in improving chronic pain, clinical symptoms related to irritable bowel syndrome, as well as insulin resistance and glycemic control in people with diabetes [3].

Mindfulness improves attention control, emotion regulation, and self-awareness, enhancing the management of lifestyles related to CVD risk factors, such as diet, physical activity, smoking, and medication adherence [6,7]. Diabetes is one of the lifestyle-related diseases that requires appropriate self-management; thus, mindfulness interventions should have an impact on treating diabetes. Indeed, there is a number of randomized controlled trials (RCTs) and systematic reviews assessing the effectiveness of mindfulness interventions on glycemic control in people with diabetes. The most recent RCT reported that a mindfulness intervention integrated into a diabetes self-management education and support program improved diabetes distress as well as hemoglobin A1c (HbA1c) levels in veterans with type 2 and type 1 diabetes [8].

Diabetes is a multifaceted and chronic disease that necessitates demanding self-management by patients. This includes organizing their diet and physical activity according to the guidance of healthcare professionals, monitoring blood glucose levels, and maintaining medication adherence. Diabetic complications, including CVD, are a major cause of morbidity and mortality among those with diabetes. Additionally, the considerable burdens of impaired functioning and self-management responsibilities can produce significant emotional distress [9]. Therefore, the beneficial effects of mindfulness on disease distress may help diabetes patients live happy lives no different from healthy individuals, which is the ultimate goal in the management of diabetes.

On the other hand, the underlying mechanism of the effects of mindfulness on physical health has not yet been clarified. Creswell et al. [3] argued that its mechanism of action consists of three pathways: the biological pathway, the health behavior pathway, and the psychological pathway. Among these, the biological pathway is vital in investigating the effect of mindfulness on glucose metabolism. The main biological effect of mindfulness is attributed to controlling the stress regulatory regions of the prefrontal cortex (the regulatory pathway) and the stress alarm system of the brain (the reactivity pathway). Previous studies have reported that mindfulness interventions are associated with a reduction in interleukin (IL)-6 levels in stressful individuals and can also modulate the autonomic nervous system (ANS) and the hypothalamic-pituitary-adrenal (HPA) axis in response to external stress [3].

The author acknowledges that yoga, qigong, and tai chi differ from mindfulness interventions in this review because these mind-body exercises involve a stronger exercise aspect compared to MBSR or MBCT. However, the mechanisms of action of mind-body therapies, such as yoga, qigong, and tai chi, are highly suggestive in considering the effects of mindfulness interventions in people with diabetes. Slow breathing and meditation reduce sympathetic nerve activity and enhance parasympathetic nerve activity, balancing the ANS and leading to stable glycemic control [10]. Furthermore, yoga practice may improve cardiovascular health by producing adiponectin and endothelial nitric oxide, and have an anti-inflammatory effect on IL-6 and tumor necrosis factor (TNF)-α [11]. Mindfulness interventions induce epigenetic modifications related to glucose metabolism and inflammation, including DNA methylation [12]. For example, García-Campayo et al. [13] performed a genome-wide screening of DNA methylation in peripheral blood leukocytes and found that there were 64 differentially methylated regions corresponding to 43 genes related to glucose and lipid metabolism in individuals who usually practice mindfulness meditation. They also identified TNF and nuclear factor kappa light chain enhancer of activated B cells (NF-κB) signaling as vital regulators of genes related to mindfulness interventions. The growing evidence suggests that mindfulness benefits physical health, including glycemic control, by modulating the ANS, HPA axis, and pro-inflammatory cytokines.

This review aims to summarize the current evidence of the impact of mindfulness interventions on glycemic control in people with diabetes and propose suggestions for future research.

## 2. Methods

This overview adhered to the guidelines of Preferred Reporting Items for Systematic Reviews and Meta-Analyses (PRISMA) (Supplementary file). The protocol for this overview was not registered in a database such as PROSPERO before conducting the literature search. The author searched the literature on mindfulness using MEDLINE/PubMed, Embase, and Cochrane Library from their inception to May 2023. The search terms for the overview were “mindfulness” OR “meditation” AND “diabetes” AND “systematic review”. The eligibility criteria were as follows: (1) Systematic review with meta-analysis of RCTs; (2) Subjects are people with type 2 and/or type 1 diabetes; (3) Interventions are mindfulness or mindfulness-based meditation; (4) Study outcomes include parameters related to glycemic control.

The initial search yielded 203 systematic reviews. Of these, 76 articles were excluded because their subjects were not diabetes patients. Additionally, 112 articles related to diet, exercise, and drug therapy were excluded. Fifteen articles were assessed for eligibility, and eight articles were excluded because they were qualitative analyses. The titles and abstracts of the screened articles were reviewed to determine their relevance. Additionally, three full-text articles that did not meet the eligibility criteria were excluded. A total of five systematic reviews were included.

The author utilized the Joanna Briggs Institute data extraction guide [14] to extract information from the included systematic reviews and meta-analyses. The methodological quality of the meta-analysis was assessed using A Measurement Tool to Assess Systematic Reviews version 2 (AMSTAR 2) [15]. 

**Figure 1:**
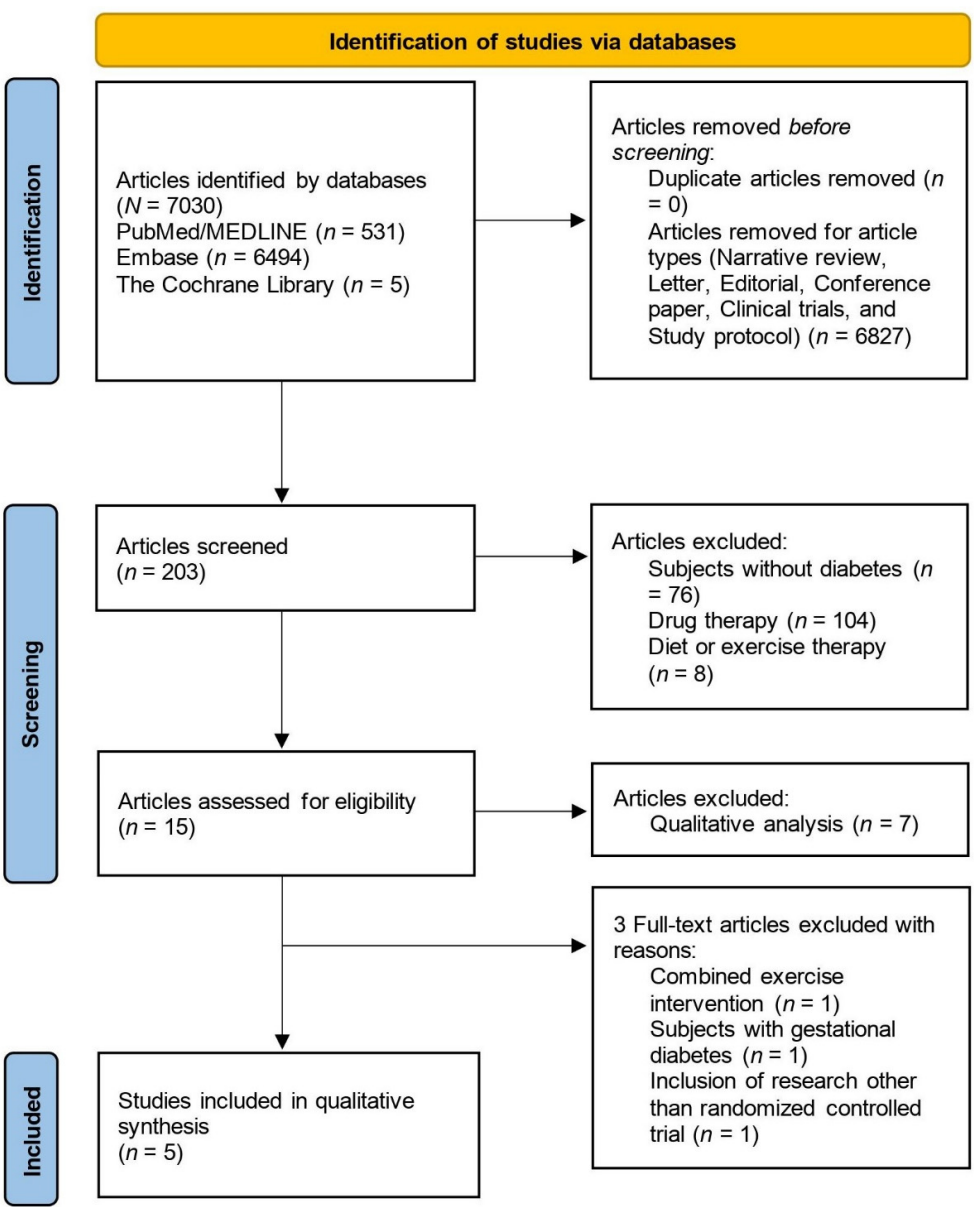
Flow diagram of the study identification, inclusion, and exclusion.

Figure 1 depicts the identification, inclusion, and exclusion procedures of this overview.

## 3. Results

### 3.1. Effects of Mindfulness Interventions on Glycemic Control

The primary outcomes of the previous studies assessing the effects of mindfulness interventions on health in people with diabetes were mostly psychological outcomes. Ni et al. [16] assessed the effectiveness of MBSR and MBCT on depression, quality of life, and HbA1c levels in people with type 2 or type 1 diabetes. The authors included nine RCTs (11 articles) with 741 subjects in the meta-analysis [17,18,19,20,21,22,23,24,25,26,27]. This systematic review reported that MBSR or MBCT significantly reduced HbA1c levels (mean difference (MD) = −0.28%; 95% confidence interval (CI) −0.47 to −0.09, *p* = 0.004) with no heterogeneity between the included studies (*I*^2^ = 0%). However, three RCTs conducted in China [24,25,26,27,28] and one RCT conducted in Canada [23] did not perform intention-to-treat analysis. The authors also did not disclose conflicts of interest and funding sources in their paper. 

The same research group reported beneficial effects of mindfulness interventions on glycemic control and psychological outcomes in people with diabetes in another academic journal [28]. This systematic review included eight RCTs with 841 subjects (11 articles) [17,18,19,20,21,22,23,29,30,31,32]. The differences between the authors’ previous and present systematic review were as follows. First, the authors excluded studies published in Chinese. Second, the authors included studies using mindfulness-based eating awareness training as a mindfulness intervention in addition to MBSR and MBCT. The pooled analysis showed that mindfulness interventions significantly reduced HbA1c levels (MD = −0.25%; 95% CI, −0.43 to −0.07, *p* = 0.006) with no heterogeneity between the included studies (*I*^2^ = 0%). Subgroup analysis also showed that subjects with better glycemic control at baseline (HbA1c levels < 8.0%) had greater reductions in HbA1c levels (−0.26%) compared to those with poor glycemic control (HbA1c levels ≥ 8.0%) (−0.22%, non-significant within-group change). The authors evaluated the risk of bias using the Cochrane risk-of-bias tool; however, other quality assessments were not performed to improve the certainty of the evidence.

Ngan et al. [33] examined the effects of MBSR, MBCT, and acceptance-based interventions, which are a form of cognitive behavioral therapy (CBT) that aims to alleviate psychological distress and discomfort by enhancing skills related to acceptance, on diabetes distress and HbA1c levels in people with type 2 diabetes. A total of nine RCTs with 801 subjects were included in the meta-analysis [20,21,31,32,34,35,36,37,38]. HbA1c levels were significantly reduced up to one month after the interventions (MD = −0.35%; 95% CI, −0.67 to −0.04, *p* = 0.03) with no heterogeneity between the included studies (*I*^2^ = 0%); however, this beneficial effect disappeared at three to six months after the interventions. This systematic review evaluated the methodological quality and the certainty of the evidence of the included studies using the Cochrane risk-of-bias tool for randomized trials ver.2 (RoB 2.0) [39] and the Grading of Recommendations Assessment, Development and Evaluation (GRADE) criteria [40], respectively, leading to the rigorous assessment of the quality of RCTs. However, the author could not identify the protocol of this systematic review in an international prospective register of systematic reviews, such as PROSPERO and Cochrane Collaboration. Moreover, although acceptance-based interventions are related to mindfulness, emphasizing the cultivation of awareness to accept present circumstances and emotions [41], the author has unresolved questions regarding whether it is appropriate to recognize mindfulness and acceptance-based interventions in a similar manner.

Heo et al. [42] assessed the effects of meditation on self-management in people with type 2 diabetes. A total of nine RCTs (10 articles) with 698 subjects were included in the analysis [17,19,31,32,36,43,44,45,46,47]. The meta-analysis revealed that meditation, including MBSR, MBCT, and mindfulness meditation, improved HbA1c levels (effect size = −0.75; 95% CI, −1.30 to −0.21, *p* = 0.007). The mean difference in HbA1c levels was −0.73 ± 0.63% in the intervention group and −0.12 ± 0.28% in the control group, respectively. However, the heterogeneity between the included studies was high (*I*^2^ = 93.2%). On the other hand, there was no significant effect on fasting blood glucose levels. The effect of meditation on lifestyle (e.g., diet, exercise), blood pressure, and blood cholesterol levels was inconclusive because the quantitative analysis was not performed.

In contrast, Bersch-Ferreira et al. [48] could not find a beneficial effect of mindfulness practice on glycemic control in people with type 2 diabetes. Only four RCTs [17,30,32,36] with 322 subjects were included in their systematic reviews. Mindfulness interventions, including MBSR and mindfulness-based eating awareness training, did not reduce blood glucose levels (two RCTs; MD = −0.73 mg/dL; 95% CI, −10.49 to 9.02) and HbA1c levels (two RCTs; MD = 0.05%; 95% CI, −0.22 to 0.32). The quality of evidence as evaluated by the GRADE system was very low for blood glucose levels and low for HbA1c levels, respectively. The authors concluded that mindfulness interventions have no effect on glycemic control in type 2 diabetes patients.

Table 1 summarizes the results of these systematic reviews. 

Overall, the quality of these systematic reviews and meta-analyses is low to moderate for the following reasons: (1) the review authors did not adequately describe the characteristics of study subjects, such as metabolic parameters and medication, which are essential for assessing glycemic control in people with diabetes in the included RCTs; (2) the authors did not refer to the sources of funding for the studies included in the reviews.

Table 2 presents the quality of systematic reviews and meta-analyses included in the overview based on AMSTAR 2 criteria.

### 3.2. Other Effects of Mindfulness Interventions in People with Diabetes

The author briefly mentions other health effects of mindfulness interventions that were confirmed in the previous systematic reviews. Three out of four systematic reviews assessed the effects of mindfulness interventions on psychological outcomes. Ni et al. [16] showed that MBSR or MBCT reduced depression scores measured by various scales (standardized mean difference (SMD) = −0.84; 95% CI, −1.16 to −0.51, *p* < 0.0001) and improved the mental health composite of quality of life (MD = 7.06, 95% CI, 5.19 to 9.03, *p* < 0.0001). Ni et al. [28] also reported that mindfulness interventions reduced depression (SMD = −0.56; 95% CI, −0.82 to −0.30, *p* < 0.0001), stress (SMD = −0.53; 95% CI, −0.75 to −0.31, *p* < 0.0001), and diabetes-related distress (MD = −5.81; 95% CI, −10.10 to −1.52, *p* = 0.008). However, the effect of mindfulness interventions on anxiety was inconsistent among the included studies. Two RCTs reported a significant reduction in anxiety in the intervention group compared to the control group, while three RCTs found no significant differences between groups. Moreover, Ngan et al. [33] showed that MBCT and self-directed mindfulness practice reduced anxiety (SMD = −0.41; 95% CI, −0.66 to −0.15, *p* = 0.002); however, self-management was not improved by the interventions. Additionally, no significant improvements in acceptance of diabetes, understanding of diabetes, and diabetes treatment satisfaction were observed.

## 4. Discussion

This review demonstrated that mindfulness interventions improved glycemic control in people with type 2 diabetes. Overall, the quality of the included systematic reviews and meta-analyses was rated as low to moderate. Two out of the five studies did not clarify the protocol of the systematic review in an international prospective register of systematic reviews. Moreover, most of the reviews did not provide adequate details about the included RCTs. None of the systematic reviews reported the sources of funding for the included RCTs. Unfortunately, the assessment of publication bias was not possible due to the limited number of included RCTs. These issues likely increased the risk of bias in the included systematic reviews and meta-analyses. There are also significant limitations regarding the interpretation of the results.

First, a standardized mindfulness intervention does not exist. MBSR and MBCT are widely used as interventions in previous studies; however, the difference between conventional CBT and mindfulness interventions remains ambiguous in terms of their definition. The study by Ngan et al. [33] included acceptance and commitment therapy, which is similar to CBT, in mindfulness interventions. On the other hand, the study by Ni et al. [26] described CBT as one of the comparators. To distinguish mindfulness interventions from psychotherapeutic approaches, including CBT, it is important to explore and clarify the differences in their impact on physiological and biochemical factors, not just psychological indicators measured using subjective methods. For instance, a meta-analysis reported that yoga practice was associated with the improvement in regulating ANS and HPA axis, indicated by decreased ambulatory blood pressure, resting heart rate, and cortisol levels [49]. Mindfulness interventions may also significantly reduce cortisol, IL-6, and TNF-α levels in depressed individuals [50]. Additionally, mindfulness meditation could probably decrease NF-kB activity and circulating C-reactive protein levels, increase T helper cell count in individuals with HIV, and increase telomerase activity [51]. Future research should explore the differences in biological impacts between mindfulness interventions and various psychotherapeutic approaches on physical functions in humans, such as cardiovascular, endocrine, immune, and central nervous system functioning. It is essential to clarify the distinctions between these interventions in terms of their effects.

Furthermore, the delivery method of mindfulness interventions was heterogeneous in previous studies. Most studies delivered the sessions in person; however, some studies used individual sessions while others used group sessions. In addition, interventionists and supervisors varied depending on the studies: certified instructors, healthcare professionals with training, psychologists, and researchers. The effectiveness of mindfulness interventions could be affected by such differences. As the review authors mentioned the limitation of their study, future research should focus on standardizing the intervention methods, including the delivery method (who and how), the number and duration of sessions, and the overall duration of interventions. This standardization should be based on a consensus derived from scientific evidence.

Third, it is imperative that researchers investigate the influence of drug therapy on study outcomes in people with diabetes. However, the systematic reviews included in this review did not report or analyze the interaction between the effects of mindfulness interventions and drugs, such as oral hypoglycemic agents. Furthermore, the systematic review should describe how the included RCTs deal with the diet and exercise of study subjects. Mindfulness interventions should affect stress-related health behaviors. Several RCTs have suggested that mindfulness interventions can alter eating behaviors, such as binge eating [52] and sweet consumption [53], and increase self-reported moderate-to-vigorous physical activity [54]. Thus, future systematic reviews and meta-analyses should take into consideration the impact of diet, exercise, and drug therapy when assessing the effect of mindfulness interventions on glycemic control in people with diabetes.

Fourth, the included systematic reviews lack some essential information on study subjects. People with diabetes often have comorbid obesity; thus, indices of obesity, such as body mass index or body weight, should be described in the systematic reviews. HbA1c levels of study subjects at baseline, which were used as an indicator of the disease condition in diabetes patients, should also be presented in summary tables. Although the study by Ni et al. [28] performed a subgroup analysis dividing the included studies into two subgroups based on baseline HbA1c levels (<8% or ≥8%), specific HbA1c levels should be shown as critical information in all the included studies. Additionally, HbA1c levels are average blood glucose levels for the last two to three months. Thus, the results of the studies with a duration of less than 12 weeks could be unreliable. Furthermore, the status of diabetic complications in study subjects is completely unknown. Uncontrolled diabetes can cause vascular damage, typically resulting in neuropathy, retinopathy, and nephropathy over time. Most people with diabetes have at least one complication, and CVD due to uncontrolled diabetes is one of the major causes of morbidity and mortality in these patients [55,56]. Such severe vascular complications significantly impair the physical function of people with diabetes. The effects of mindfulness interventions on glycemic control or other health outcomes in patients with progressed diabetic complications remains unknown based on the systematic reviews and meta-analyses included in this review. Therefore, future research should investigate whether mindfulness interventions provide health benefits to those patients.

Fifth, previous studies did not seem to investigate the effects of mindfulness interventions on other parameters related to glucose metabolism, such as insulin sensitivity. A recent study showed that adolescents who exhibit lower levels of mindfulness and experience shorter sleep durations have a higher risk of developing insulin resistance, whereas higher levels of mindfulness may serve as a protective factor [57]. Inflammation, insulin resistance, and leptin resistance appear to be key risk factors for the development of depression and anxiety, which mindfulness interventions could potentially benefit [58]. Future studies should investigate the endocrinological mechanisms that underlie the relationship between mindfulness and glucose metabolism to elucidate the impact of mindfulness interventions on diabetes.

Finally, the systematic reviews in this review did not evaluate the publication bias since the number of included studies in the meta-analyses was less than 10. The Cochrane Handbook for Systematic Reviews of Interventions suggests a minimum of 10 studies per examined covariate in meta-analysis [59]. Therefore, a future systematic review should include at least 10 high-quality RCTs to obtain rigorous evidence on whether mindfulness interventions have specific health benefits in people with diabetes.

The RCTs included in previous systematic reviews exhibited significant heterogeneity attributed to differences in the characteristics of study subjects, study duration/follow-up period, sample size, and outcomes. Therefore, high-quality RCTs with larger sample sizes and longer durations/follow-up periods are warranted.

Although it might be beyond the scope of this overview, future research should focus on the gut-brain interaction and epigenetics. The gut-brain axis demonstrates bidirectional communication between the central nervous system and gastrointestinal function, including the ANS function, HPA axis, neuroendocrine system, immune system, and gut microbiota [60]. The bidirectional microbiota-gut-brain axis connects stress and depression in both directions: stress influences the microbiota through the efferent brain-gut axis, and the gut microbiota, in turn, affects the central nervous system through the afferent gut-brain axis. Thus, mindfulness interventions could reduce stress, modulate the gut microbiota, and regulate endocrinological and immune functions, benefiting physical health [61]. On the other hand, the gut microbiota has been associated with the development of obesity, metabolic syndrome, and type 2 diabetes due to its impact on reduced glucose tolerance and insulin sensitivity, and gut microbiota dysbiosis is associated with inflammation and the progression of diabetic complications in people with type 2 diabetes [62,63]. The gut microbiota may be the key to elucidate the mechanisms of action of mindfulness interventions in glycemic control. Recently, mindfulness practice has also been shown to have a beneficial impact on epigenetic changes by regulating the HPA axis, serotonergic, metabolic, immunological, and neurological pathways [64]. Future clinical studies are required to explore these biological factors in addition to glycemic control in people with diabetes.

This overview also had some limitations. First, it was conducted by a single author, which may introduce biases in the literature search, eligibility screening, and data extraction processes. Second, quantitative analysis of the results from previous systematic reviews and meta-analyses was not conducted. To ensure higher-quality conclusions, it will be necessary to incorporate both qualitative and quantitative analysis in future studies.

## 5. Conclusions

Previous systematic reviews and meta-analyses have shown that mindfulness interventions are effective in improving glycemic control in people with type 2 diabetes. The reduction in HbA1c levels is approximately 0.3%, which somewhat limits the clinical significance in managing people with type 2 diabetes. Nevertheless, mindfulness interventions could effectively complement diet and exercise therapy for people with type 2 diabetes. On the other hand, mindfulness interventions had no impact on fasting blood glucose levels. Mindfulness interventions could potentially be beneficial in reducing stress, depression, and anxiety in people with diabetes. However, it should be noted that the quality of the evidence supporting this claim was not high. These health benefits are partly due to the regulation of ANS function and the HPA axis, as well as behavioral changes facilitated by mindfulness. However, the effects of mindfulness interventions on cardiovascular, endocrine, and central nervous system function are not fully clarified. The beneficial effect of mindfulness interventions on glycemic control in people with diabetes is inconclusive due to the suboptimal quality of previous systematic reviews and meta-analyses. Mindfulness could be a feasible and supportive practice for people with diabetes who also suffer from stress, depression, and anxiety; however, more rigorous scientific evidence will be needed in the future.

## Figures and Tables

**Table 1:** Systematic reviews and meta-analyses assessing the effects of mindfulness interventions on people with diabetes.

Authors, Year	Subjects	Interventions	Comparators	Outcomes	Results
Ni et al., 2020 [16]	People with type 2 or type 1 diabetes Age: 18.0–68.7 years in the intervention group; 18.5–59.8 years in the control group Sex (male): 44–78.2%	MBSR or MBCT Method of conducting sessions: Face-to-face (individual or group), video group Duration of sessions: 45–180 min/session Number of sessions: 6–9 sessions Duration of interventions: 2–9 weeks	Usual care, wait-list, no intervention, or health education	HbA1c, QoL, depression	HbA1c levels ↓ QoL ↑ Depression ↓
Ni et al., 2021 [28]	People with type 2 or type 1 diabetes Age: No description Sex (male): 36.6–78.2%	MBSR, MBCT, MB-EAT, or unspecific mindfulness-based intervention Method of conducting sessions: Face-to-face (individual or group), using audio compact disc at home Duration of sessions: 30–150 min/session Number of sessions: 8–9 sessions Duration of interventions: 8 weeks–3 months	Usual care, walking, patient education, diabetes self-management education-based intervention, wait-list, CBT	HbA1c, stress, depression, anxiety, distress	HbA1c levels ↓ Diabetes-related distress ↓ Stress ↓ Depression ↓ Anxiety → (based on a qualitative synthesis)
Ngan et al., 2020 [33]	People with type 2 or type 1 diabetes Age: 53.8–66.3 years (mean) Sex: No description	MBSR, MBCT, acceptance and commitment therapy, or self-directed mindfulness practice Method of conducting sessions: Face-to-face (individual or group), using audio compact disc at home Duration of sessions: 30–420 (one day workshop) min/session Number of sessions: 1–10 sessions Duration of interventions: One day (7-h workshop)–10 weeks	Diabetes education, usual care, annual routine visit, no intervention	HbA1c, diabetes distress, diabetes self-management, psychological symptoms	HbA1c levels ↓ Diabetes distress ↓ Self-management → Anxiety ↓ Depression ↓
Heo et al., 2023 [42]	People with type 2 diabetes Age: 42.1–78.9 years (mean) Sex (male): 0–78.2%	MBSR, MBCT, or mindfulness meditation program Method of conducting sessions: Face-to-face (individual or group), using audio compact disc at home Duration of sessions: 30–150 min/session Number of sessions: 8–10 sessions Duration of interventions: 8–10 weeks	Usual care, diabetes education, wait-list, walking	Blood glucose levels, HbA1c, self-management (e.g., diet, exercise, blood pressure, cholesterol, obesity, and foot care)	HbA1c levels ↓ Fasting blood glucose levels → Self-management (unknown)
Bersch-Ferreira et al., 2021 [48]	People with type 2 diabetes Age: 53.5–68.5 years (mean) Sex (male/female): 188/134	MBSR and MB-EAT Method of conducting sessions: Face-to-face (individual or group) Duration of sessions: 30–150 min/session Number of sessions: 6–8 sessions Duration of interventions: 8–12 weeks	Usual care, diabetes education, walking, no intervention	Blood glucose levels, HbA1c	HbA1c → Blood glucose levels →

MBSR, mindfulness-based stress reduction; MBCT, mindfulness-based cognitive therapy; MB-EAT, mindfulness-based eating awareness training; QoL, quality of life; HbA1c, hemoglobin A1c; CBT, cognitive behavioral therapy; ↑, increase or improve; →, no change; ↓, decrease.

**Table 2:** Quality assessment of studies included in the overview using A Measurement Tool to Assess Systematic Reviews (AMSTAR) 2.

	Ni et al., 2020 [16]	Ni et al., 2021 [28]	Ngan et al., 2020 [33]	Heo et al., 2023 [42]	Bersch-Ferreira et al., 2021 [48]
1. Did the research questions and inclusion criteria for the review include the components of PICO?	Yes	Yes	Yes	Yes	Yes
2. Did the report of the review contain an explicit statement that the review methods were established prior to conducting the review and did the report justify any significant deviations from the protocol?	Yes	Yes	Unclear	Unclear	Yes
3. Did the review authors explain their selection of the study designs for inclusion in the review?	Yes	Yes	Yes	Yes	Yes
4. Did the review authors use a comprehensive literature search strategy?	Yes	Yes	Yes	Yes	Yes
5. Did the review authors perform study selection in duplicate?	Yes	Yes	Yes	Yes	Yes
6. Did the review authors perform data extraction in duplicate?	Yes	Yes	Yes	Yes	Yes
7. Did the review authors provide a list of excluded studies and justify the exclusions?	Yes	Yes	Yes	Yes	Yes
8. Did the review authors describe the included studies in adequate detail?	No	No	No	No	Partly Yes
9. Did the review authors use a satisfactory technique for assessing the risk of bias (RoB) in individual studies that were included in the review?	Yes	Yes	Yes	Yes	Yes
10. Did the review authors report on the sources of funding for the studies included in the review?	No	No	No	No	No
11. If meta-analysis was performed, did the review authors use appropriate methods for statistical combination of results?	Yes	Yes	Yes	Yes	Yes
12. If meta-analysis was performed, did the review authors assess the potential impact of RoB in individual studies on the results of the meta-analysis or other evidence synthesis?	Yes	Yes	Yes	Yes	Yes
13. Did the review authors account for RoB in individual studies when interpreting/discussing the results of the review?	Yes	Yes	Yes	Yes	Yes
14. Did the review authors provide a satisfactory explanation for and discussion of any heterogeneity observed in the results of the review?	Yes	Yes	Yes	Yes	Yes
15. If they performed quantitative synthesis, did the review authors carry out an adequate investigation of publication bias (small study bias) and discuss its likely impact on the results of the review?	Unassesable	Unassesable	Unassesable	Unassesable	Unassesable
16. Did the review authors report any potential sources of conflict of interest, including any funding they received for conducting the review?	No	Yes	Yes	Yes	Yes
**Rating result**	Low	Moderate	Low	Low	Moderate

## Data Availability

Not applicable.

## Supplementary Materials

The following supporting information can be downloaded at: https://www.mdpi.com/article/10.3390/medicines10090053/s1, PRISM Checklist [65].
